# The Urine, the Common Poisons, and the Milk

**Published:** 1891-11

**Authors:** 


THE URINE, THE COMMON POISONS, AND THE MILK. By J. W. Holland, M. D., Professor of Chemistry and Toxicology, Jefferson Medical College: Published by P. Blakiston, Son & Co., Philadelphia.

This most excellent little work is out in a fourth edition, revised, enlarged and illustrated. The text is clear and to the point and goes quite thoroughly into the examination of the urine, giving well-printed cuts of the microscopical appearance of the normal and pathological constituents found in the urine. The ordinary qualitative tests for urinary ingredients are printed in large type so that the busy practitioner and student whose time is short, can readily turn to them. The explanations and qualitative processes are given in small type, so that those who wish may make a more thcrough study of the subject. The book is small, not too large to carry in the pocket, and is arranged with blank pages for notes, memoranda, etc., thus making it a very desirable work for the practical student engaged in actual laboratory work. L. H. J.

### PILOCARPINE IN PUERPERAL ECLAMPSIA.

In the *Gazette hebdomadaire des sciences medicales* for September 12th, Dr. Strisover adds to the experience of observers in this field the results of his use of pilocarpine in the treatment of eclampsia. By the subcutaneous injection of hydrochloride of pilocarpine the author has been successful in controlling the convulsions and preventing their recurrence in ten cases. The treating successively of such a number of cases without one death has led the author to the conclusions that pilocarpine is an antagonist to the eclamptic process; that feebleness of the pulse is not a contra-indication to the repeated injection of the drug, so long as the convulsions reappear; and, finally, that the condition of the pupils is to be relied npon as an index to the further accession of the convulsions or to immunity by the physiological action of the drug.—*N. Y. Med. Journal.*

### PERIODS OF GESTATION.

The periods of gestation are the same in the horse and ass, eleven months each; camel, twelve months; elephant, two years; lion, five months; buffalo, twelve months; cow, nine months; sheep, five months; reindeer, eight months; monkey, seven months; bear, six months; sow, four months; dog, nine weeks; cat, eight weeks; rabbit, four weeks; guinea pig, four weeks; wolf, ninety to ninety-five days. Goose sets thirty days; swans, forty-two days; hens twenty-one days; ducks, twenty-eight days; pea hens and turkeys, twenty-eight days; canaries, fourteen days; pigeons, fourteen days; parrots, forty days.—Ex.

